# An Engineered Glove for the Objective Assessment of Hand Dexterity in Patients With Systemic Sclerosis

**DOI:** 10.1002/acr2.90082

**Published:** 2026-07-03

**Authors:** Alberto Sulli, Elvis Hysa, Paolo Clini, Emanuele Gotelli, Tamara Vojinovic, Carmen Pizzorni, Ali Jaffal, Sabrina Paolino, Rosanna Campitiello, Vanessa Smith, Maurizio Cutolo

**Affiliations:** ^1^ Laboratory of Experimental Rheumatology and Academic Division of Clinical Rheumatology, Department of Internal Medicine University of Genova Genova Italy; ^2^ IRCCS A.O.M. Ospedale Policlinico San Martino Genova Italy; ^3^ Department of Experimental Medicine University of Genova Genoa Italy; ^4^ Department of Internal Medicine Ghent University Ghent Belgium; ^5^ Department of Rheumatology Ghent University Hospital Ghent; ^6^ Unit for Molecular Immunology and Inflammation, Vlaams Instituut voor Biotechnologie Inflammation Research Center Ghent Belgium

## Abstract

**Objective:**

Hand disability is a major musculoskeletal functional impairment in systemic sclerosis (SSc), but its objective assessment remains challenging. The validated Hand Test System (HTS) engineered glove has provided quantitative dexterity data in rheumatic diseases. The primary objective was to evaluate the applicability of the HTS glove in patients with SSc by comparing its parameters (touch duration [TD], intertapping interval [ITI], and movement rate [MR]) with healthy controls (HCs). The assumptions were that HTS parameters would be significantly impaired in patients with SSc and would correlate with clinical, imaging, and patient‐reported outcome measures (PROMs).

**Methods:**

A cross‐sectional study was conducted enrolling 25 patients with SSc (fulfilling 2013 American College of Rheumatology/EULAR criteria) and 25 matched HCs. The main outcome variables were the HTS glove parameters. All participants also underwent grip strength measurement and an assessment of PROMs (Scleroderma Health Assessment Questionnaire and Duruöz Hand Index), together with a performance‐based evaluation of hand mobility using the Hand Mobility in Scleroderma test. Clinical and imaging data were collected for patients with SSc including the modified Rodnan skin score (mRSS) and high‐frequency skin ultrasonography (HFSU) to measure hand and total body dermal thickness.

**Results:**

Patients with SSc performed finger movements significantly more slowly and less efficiently than HCs, demonstrated by prolonged mean TD and ITI and reduced MR (*P* < 0.05 for all). In patients with SSc, HTS parameters significantly correlated with poorer PROMs (0.39 < r < 0.65, all *P* < 0.01 for all). Notably, both mean TD and ITI directly correlated with the mRSS (0.40 < r < 0.48, all *P* < 0.05). Furthermore, the mean TD showed a significant direct correlation with HFSU‐measured dermal thickness of the dorsum of the hands and with total body dermal thickness (*P* < 0.05 for all). No significant differences in HTS parameters were detected based on nailfold videocapillaroscopy findings or organ involvement.

**Conclusion:**

The HTS glove provides objective measures of the impaired hand dexterity in SSc. The significant correlations between HTS parameters, PROMs, mRSS, and HFSU‐measured dermal thickness suggest a significant relationship between skin fibrosis and functional hand disability. The HTS glove seems a promising tool for objectively monitoring SSc hand outcomes, and studies to explore its utility in monitoring therapeutic responses are in progression.

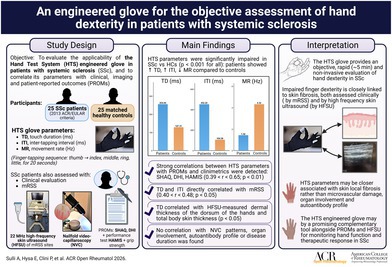

## INTRODUCTION

Systemic sclerosis (SSc) is a rare systemic autoimmune connective tissue disorder characterized by immune system dysregulation, leading to vasculopathy and fibrosis of the skin and internal organs, causing significant morbidity and mortality.^1^ It predominantly affects women (4:1 ratio), with a prevalence ranging from 50 to 300 cases per million people.

SSc presents with a variety of symptoms affecting multiple organ systems, and hand disability is a common musculoskeletal manifestation significantly impacting patient's quality of life, which may arise from several causes including Raynaud phenomenon, swollen hands and/or sclerodactyly, digital ulcers, tendon friction rub, and progressive joint contractures.^1^, ^2^ Nailfold capillary abnormalities are present in all patients with SSc and they represent an important imaging biomarker of the disease severity itself.^3^, ^4^, ^5^

Assessing hand function in SSc is challenging and sensitive tools are needed beyond clinimetric indexes. Although traditional clinimetric indexes and patient‐reported outcome measures (PROMs) provide valuable clinical insights, they frequently rely on subjective patient perception or semiquantitative evaluator scoring. Consequently, these traditional tools can be susceptible to recall bias, inter‐rater variability, and ceiling or floor effects. Novel wearable instruments are therefore highly useful, as they may bypass these limitations by objectively capturing subtle, real‐time kinematic alterations in fine motor control that standard questionnaires or visual assessments might overlook. Data gloves, also known as sensor gloves or smart gloves, are wearable devices equipped with sensors that track and capture hand and finger movements in real time. These gloves are classified based on the type of sensors they use, each of which serves different purposes and is suitable for various medical applications.^6^

Medical‐grade data gloves typically include multiple sensors along each finger to detect even the smallest movements and gestures, and are made from lightweight, breathable materials to ensure comfort during use.^7^

The Hand Test System (HTS) glove is an innovative and validated sensor‐engineered device, designed for the precise evaluation of hand function.^8^ It was found able to provide detailed quantitative data on hand mobility in patients with rheumatoid arthritis (RA).^9^, ^10^

In particular, the HTS glove showed a good sensitivity in detecting the dexterity of the finger opposition movements, and the glove parameters showed statistically significant correlations with clinimetric and clinical indexes in patients with RA as cross‐sectionally as during the follow‐up.^9^, ^10^ The HTS glove evaluates hand dexterity through three primary quantitative parameters: touch duration (TD), defined as the average contact time between fingers; intertapping interval (ITI), representing the average time between consecutive touches; movement rate (MR), which reflects the overall frequency of touches per second. Clinically, these parameters directly translate to a patient's ability to execute activities of daily living (ADLs). For instance, an increased TD implies difficulty and stiffness in releasing a pinch grip, whereas an increased ITI and decreased MR indicate delayed motor planning and a general loss of agility. Functionally, a patient presenting with these altered parameters will likely experience significant difficulty performing fine‐motor ADLs, such as buttoning a shirt, tying shoelaces, typing, or manipulating small household objects. Beyond its validation in RA, the HTS glove has also been successfully studied in the field of neurology to assess hand and finger involvement in patients with multiple sclerosis and Charcot‐Marie‐Tooth neuropathy.^8^, ^11^ However, to our knowledge, it has not yet been investigated in any other autoimmune rheumatic disease apart from RA.

The aim of the present study was to test the HTS glove in patients with SSc, in comparison with healthy controls (HCs) and to measure hand dexterity, as well as to correlate HTS glove parameters with disease‐related measures, including clinical scores, imaging‐derived findings from nailfold capillaroscopy (NVC), and high‐frequency skin ultrasonography (HFSU).

## PATIENTS AND METHODS

### Population

Twenty‐five patients with SSc were enrolled at the Rheumatologic Outpatient Clinic from February 2024 to September 2024. SSc was diagnosed according to 2013 American College of Rheumatology/EULAR criteria.^12^ Twenty‐five HCs of similar age and sex were also enrolled.

Demographic and clinical features of the enrolled patients are reported in Tables 1 and 2.

**Table 1:** Clinical features of both patients with SSc and HCs enrolled in the study*

	SSc	HCs	*P* value
Demographic data			
Age, mean ± SD, y	60 ± 13	55 ± 10	0.15
Sex, female/male	22/3	20/5	0.70
Disease duration, mean ± SD, y	17 ± 13	NA	NA
Dominant hand, n (%)			
Right‐handed	25 (100)	23 (92)	0.49
Left‐handed	0 (0)	2 (8)	1
Ambidextrous	0 (0)	0 (0)	NA
Capillaroscopic patterns, n (%)			
Normal pattern	0 (0)	25 (100)	<0.001
Nonspecific alterations	4 (16)	0	0.11
“Early” pattern	2 (8)	0	0.49
“Active” pattern	8 (32)	0	0.004
“Late” pattern	11 (44)	0	<0.001

* HC, healthy control; NA, not applicable; SSc, systemic sclerosis.

**Table 2:** Clinical and demographic features of the included patients with SSc subsets*

Demographic or clinical feature	lcSSc, n = 17	dcSSc, n = 8^a^	*P* value
Female, n (%)	16 (94)	6 (75)	0.2
Age, mean ± SD, y	67.2 ± 9.8	67.6 ± 18.8	0.97
Duration of the disease, mean ± SD, y	20.4 ± 13.6	11.0 ± 7.3	0.457
mRSS, mean ± SD	3.9 ± 2.5	13.0 ± 9.0	0.18
Patients in treatment with oral vasodilating agents, n (%)	14 (82)	7 (88)	0.752
Patients in treatment with aspirin, n (%)	11 (65)	4 (50)	0.47
Patients in treatment with DMARDs, n (%)	12 (71)	6 (75)	0.826
Patients in treatment with nintedanib, n (%)	5 (29)	2 (25)	0.82
Scl‐70, n (%)	6 (35)	4 (50)	0.47
ACA, n (%)	9 (53)	0 (0)	**0.01**
Other autoantibodies (ie, RNA pol III, Ro52 or Pm‐Scl75/100), n (%)	3 (18)	2 (25)	0.67
Pulmonary involvement, n (%)	10 (59)	8 (100)	**0.04**
Gastroesophageal involvement, n (%)	9 (53)	4 (50)	0.88
Kidney involvement, n (%)	2 (12)	2 (25)	0.39
Previous digital ulcers, n (%)	8 (47)	6 (75)	0.18
Active digital ulcers, n (%)	3 (18)	3 (38)	0.27

* Significant *P* values < 0.05 are reported in bold. ACA, anticentromere antibodies; dcSSc, diffuse cutaneous systemic sclerosis; DMARDs, disease‐modifying antirheumatic drugs; lcSSc, limited cutaneous systemic sclerosis; mRSS, modified Rodnan skin score.

^a^ Patients were classified as with dcSSc if the involvement of the skin included also arms, trunk, and thighs.

The medical history for each patient was recorded, including disease subset, autoantibody profile, and current treatment. Imaging parameters at NVC (“early,” “active,” “late” patterns),^13^, ^14^ as well as dermal thickness, measured with a 22 MHz HFSU at the modified Rodnan skin score [mRSS] sites were also collected (more details in the subsequent paragraphs).

Patients with SSc were on stable standard treatment since at least three months. Exclusion criteria included the presence of local hand conditions that could compromise finger mobility or interfere with proper glove positioning: the presence of overlapping inflammatory arthritis, symptomatic hand osteoarthritis (including Bouchard or Heberden nodules, and thumb carpometacarpal osteoarthritis), carpal tunnel syndrome, tendinous nodules, Dupuytren contracture, severe hand deformities, or recent local trauma/fractures. Participants with cognitive disorders (eg, Alzheimer disease or other forms of dementia) or functional neurologic conditions affecting motor performance (such as fibromyalgia or Parkinson disease) were also excluded.

### Clinical assessment of patients with SSc

All patients with SSc underwent clinical evaluation and laboratory and instrumental assessments, as part of their routine follow‐up within a period not exceeding three months before or after the HTS test. In our cohort, the actual mean interval between the HTS test and the mRSS assessment was 42 days, whereas the mean interval with the other clinical and imaging parameters was approximately 60 days.

All hand‐specific clinimetric tests (the Hand Mobility in Scleroderma [HAMIS] and grip strength tests), PROMs (the Scleroderma Health Assessment Questionnaire [SHAQ] and the Duruöz Hand Index [DHI]) and the engineered glove test were performed sequentially on the exact same day for each patient.

Clinical parameters included evaluation of skin involvement using the mRSS (range 0–51) to quantify cutaneous thickness, and the dichotomous (yes or no) recording of sclerodactyly, puffy fingers, telangiectasias, calcinosis, and digital ulcers. According to the extent of skin involvement, patients were categorized as having limited cutaneous or diffuse cutaneous SSc (dcSSc) disease; the latter was defined by the involvement of the skin on the arms, trunk, and thighs.^15^

Visceral involvement was documented dichotomously based on the presence of specific organ manifestations: interstitial lung disease on high‐resolution computed tomography for pulmonary involvement; elevated pulmonary arterial pressure on echocardiography and/or confirmed by right‐sided heart catheterization, when indicated by guidelines, for pulmonary arterial hypertension; manometric evidence of esophageal dysmotility for esophageal involvement; and increased renal resistive index and/or elevated serum creatinine for renal impairment.

Information on current immunosuppressive and/or vasoactive therapies, as well as comorbidities, was also collected. Detection of antinuclear antibodies was performed on HEp‐2 cells using indirect immunofluorescence (Euroimmun, Lübeck, Germany)^16^ on sera at screening dilution 1:80. Detection of extractable nuclear antigen (ENA) was performed on sera using fluorescence enzyme immunoassay method in Phadia 250 (Thermofisher). ENA test, called Symphony, is a pool of different antigens: If the test is positive, further analysis is performed to identify the specific antigens (Ro, La, Sm, RNP, Scl‐70, and Jo‐1). The detection of autoantibodies associated with scleroderma is performed on sera using a dot‐blot assay (Alphadia–Alifax) searching different antigens: Scl‐70, CENP‐A, CENP‐B, PmScl‐100, PmScl‐75, Ku, RNA‐pol III, U1‐RNP, Th/To, Fibrillarin, NOR‐90, and SS‐A 52kDa.

### Clinimetric indexes, disease‐specific disability questionnaires, and HTS glove test

All clinimetric and functional assessments were conducted and supervised by the same operator (PC), a rheumatologist who underwent dedicated, standardized training for both the clinical indices and the operation of the HTS glove software before study initiation.

The assessments were performed at ambient temperature of 22 to 23°C, allowing patients to acclimate to the room temperature. Each evaluation was performed in the absence of active Raynaud phenomenon. Patients with digital ulcers were not excluded from the study. At the time of assessment, 24% of patients had a single small active digital ulcer on the dorsal aspect of the fingers (less than 3 mm in diameter), which was properly protected during testing. Given the dorsal localization, distinct from the more frequent ischemic fingertip ulcers, these lesions did not interfere with the volar fingertip surface engaged during finger‐tapping. Additionally, all tests were conducted in an environment free from auditory and visual distractions. The following evaluation was performed in all patients with SSc and HCs.

### Questionnaires and performance tests for hand function in SSc

The functional and hand‐specific status of patients with SSc was evaluated using three validated instruments. The Health Assessment Questionnaire (HAQ) for SSc (SHAQ) is a disease‐specific adaptation of the original HAQ that assesses patients’ physical ability and functional impairment, showing strong correlations with skin involvement and overall disease severity.^17^ It evaluates eight domains of daily living (dressing, arising, eating, walking, hygiene, reach, grip, and routine activities).^17^ Furthermore, it incorporates six specific visual analog scales assessing Raynaud phenomenon, digital ulcers, gastrointestinal involvement, pulmonary involvement, pain, and the patient's global assessment of disease severity over the past week.

The DHI is a self‐reported questionnaire comprising 18 items on daily activities requiring hand function (e.g., buttoning, gripping), scored from 0 to 5, with higher scores indicating greater disability; its Italian version demonstrated excellent reliability, validity, and sensitivity to change.^18^, ^19^

Finally, the HAMIS test objectively measures hand mobility through nine performance‐based tasks assessing finger and wrist movements; it has been validated in Italian and correlates well with the degree of hand skin involvement.^20^

Together, these tools provide complementary and robust assessments of hand function and disability in patients with SSc, supporting both clinical evaluation and research applications.

### Analogic dynamometer

An analogic dynamometer (Smedley Dynamometer, Gima, Gessate, Italy) was used to assess hand grip strength. The assessment was conducted following Mathiowetz's guidelines: the patient was seated with his arm in abduction and his elbow flexed at 90°.^21^ The mean value of three consecutive measurements was used.

### HTS glove

**Figure 1:**
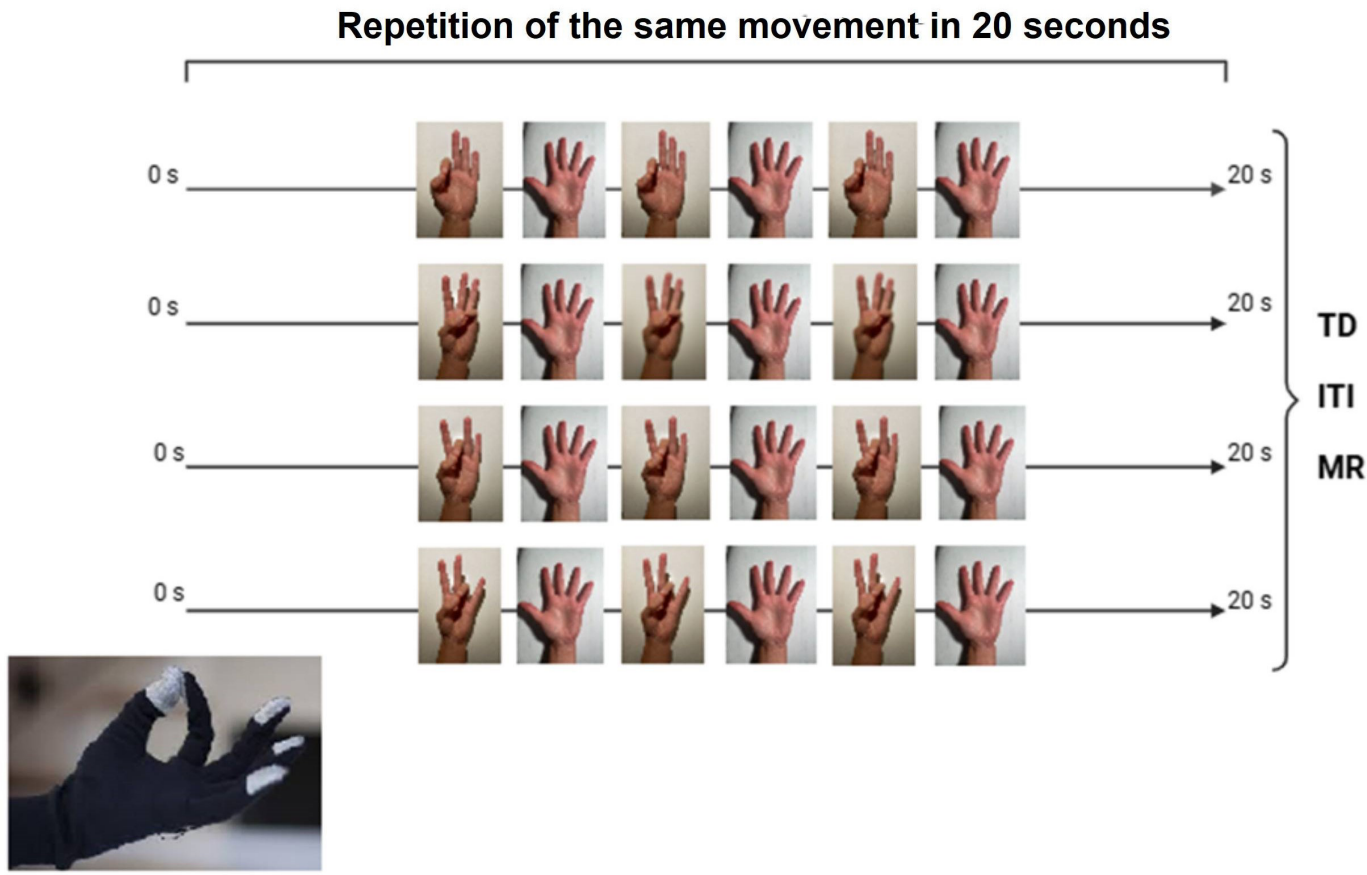
The Hand Test System glove exercise. The exercise consisted of having the patient repeat a sequence of touch as many times as possible in 20 seconds, involving the thumb and one finger at a time (thumb and index finger, thumb and middle finger, thumb and ring finger, and thumb and little finger). Image created with www.biorender.com (agreement number: ND28ZW72WX). ITI, intertapping interval; MR, movement rate; TD, touch duration.

The HTS glove is an innovative sensor‐engineered device designed for precise, objective evaluation of hand function (a photograph of the glove and a scheme of the required finger movements are provided in Figure 1). Made from elastic silk, it adapts easily to different hand sizes without restricting movement. Conductive sensors made of tin and copper are attached to the fingertips of both gloves, ensuring that hand mobility remains unaffected during use.

Through a data acquisition card (USB‐1208FS, Measurement Computing), the analyzing software provides the following quantitative three key parameters: TD, ITI, and MR, which provide quantitative data on the speed and efficiency of finger movements. TD represents the average contact time between fingers during the task, ITI measures the average time between touches, and MR reflects the frequency of touches per second.

Testing involves the patient repeating a sequence of touches as rapidly as possible for 20 seconds, using the thumb to tap each finger sequentially: index, middle, ring, and little fingers. Patients are instructed to fully extend their joints after each touch. Practice trials ensure familiarity and accuracy before formal recording. Before formal recording, patients undergo a brief familiarization period consisting of practice trials lasting approximately two to three minutes to ensure they understand the sequence and achieve full joint extension between touches. Once the patient is comfortable, the formal test is recorded, which requires the patient to repeat the touch sequence as rapidly and accurately as possible for exactly 20 seconds. The software automatically excludes incomplete or incorrect sequences from the analysis. As mentioned, data are acquired via a USB data acquisition card and processed using dedicated HTS software (see Figure 1).

### NVC

NVC is a noninvasive technique used to examine the microvascular abnormalities characteristic of SSc. The nailfolds of eight fingers, from right little to right index and from left index to left little, were examined in each patient, as previously reported using the videocapillaroscope Horus (Adamo srl).^13^, ^22^, ^23^

The following items were considered, according to the recently published NVC standardization from the EULAR Study Group on Microcirculation in Rheumatic Diseases and the Scleroderma Clinical Trials Consortium Group on Capillaroscopy: the presence of capillary dilations, giant capillaries, microhemorrhages, number of capillaries, capillary abnormal shapes or ramifications, and the proper pattern of microangiopathy (“early,”, “active,” and “late”) according to Cutolo et al was identified, as previously reported.^24^, ^25^

### HFSU

HFSU was performed using a 22 MHz linear probe (MyLab One, Esaote) in B‐mode. The examination was conducted by a single trained operator (EH) to ensure consistency and minimize inter‐rater variability. All HFSU assessments were performed at all sites included in the mRSS, following standardized protocols previously described in the literature and our prior skin mapping studies.^26^, ^27^

For each anatomical site, the probe was positioned perpendicularly to the skin surface, with a sufficient amount of ultrasound gel applied as a coupling layer to minimize compression artifacts. No additional pressure was applied beyond the probe's own weight. All examinations were performed at the same time of day, after a 15‐minute acclimatization period at a stable room temperature (22–23°C), to mitigate potential biases related to temperature variations.

Images were considered adequate when a clear distinction between the three skin layers (epidermis, dermis, and subcutis) with visible parallel interfaces was observed. Dermal thickness was measured in millimeters as the distance between the epidermis–dermis interface and the dermis–subcutis interface. For each site, the measurement was repeated three times, and the average of these three values was used for analysis.

Correlations between HFSU measurements and the engineered glove were explored for the sites on the hands, specifically the dorsum of the middle finger and the dorsum of the hand. Additionally, the total skin thickness (sum of all mRSS sites) was calculated for correlation analysis with the glove data.

### Ethical aspects

All participants provided written informed consent to enter the study and manage their clinical data, and the study was conducted in accordance with the principles of the Declaration of Helsinki and Good Clinical Practice. The Ethical Committee of San Martino Polyclinic Hospital approved this study (No. 444REG2017) and every participant provided informed consent.

### Statistics

Continuous variables were reported as mean ± SD or median with interquartile range, depending on the data distribution, whereas discrete variables were presented as count and percentage. Nonparametric tests were used for variables that were not normally distributed. Spearman's correlation test was employed to assess the relationship between glove parameters and clinical data. The Mann–Whitney U‐test was used to compare continuous variables between groups. Because both hands were assessed for each participant, interhand differences for TD, ITI, and MR were first evaluated. For the primary analyses, the mean value of the two hands was used to minimize potential bias related to hand dominance. When significant interhand differences were detected, correlations with clinical, imaging, and PROMs were subsequently performed separately for each hand. All interhand comparisons are reported in Supplementary Table 1.

### Data sharing statement

Data are available upon reasonable request.

## RESULTS

### Differences in the glove parameters between patients with SSc versus HCs

Analysis of the HTS glove parameters revealed significant differences between patients with SSc and HCs, all reaching strong statistical significance (*P* < 0.001). Specifically, both TD and ITI were significantly increased in patients with SSc, whereas MR was decreased compared to HCs, indicating impaired fine motor control (Table 3). Similarly, traditional clinimetric parameters, including the DHI, SHAQ, HAMIS, grip strength, and mRSS, were all significantly altered in patients with SSc compared to controls (see Table 3).

**Table 3:** Statistical significance differences of HTS glove parameters, as well as for standard clinimetric parameters, between patients with SSc and CNT*

	SSc	HC	Statistical significance
Clinimetric parameters, median (IQR)			
DHI	5 (25.5)	0 (0)	*P* ≤ 0.001
SHAQ	5 (25.25)	0 (0)	*P* ≤ 0.001
HAMIS	3 (6.75)	0 (0)	*P* ≤ 0.001
Grip strength	45 (22.75)	63.5 (36.25)	*P* ≤ 0.001
mRSS	5 (6.25)	0 (0)	*P* ≤ 0.001
HTS glove parameters, median (IQR)			
TD, ms	254.84 (197.60)	127.31 (35.05)	*P* ≤ 0.001
ITI, ms	452.22 (242.15)	104.56 (30.71)	*P* ≤ 0.001
MR, Hz	1.42 (0.83)	4.32 (0.68)	*P* ≤ 0.001

* DHI, Duruöz Hand Index; HAMIS, Hand Mobility in Scleroderma; HC, healthy control; HTS, Hand Test System; IQR, interquartile range; ITI, intertapping interval; MR, movement rate; mRSS, modified Rodnan skin score; SHAQ, Scleroderma Health Assessment Questionnaire; SSc, systemic sclerosis; TD, touch duration.

### Interhand comparison

The parameters TD, ITI, and MR were measured for both hands in all participants. Interhand differences are shown in Supplementary Table 1. Statistically significant differences were observed for TD in patients with SSc and for all the parameters in HCs (all *P* < 0.05). For these reasons, to minimize potential bias from hand dominance, the mean value of the two hands was used for all the primary analyses. Patterns of correlation with clinical, imaging, and PROMs data were consistent even when analyzed separately by hand for TD in patients with SSc.

### Correlations between HTS parameters and clinical or imaging findings in patients with SSc

HTS‐derived metrics (TD, ITI, and MR) demonstrated strong correlations with clinimetric indexes, including SHAQ, DHI, and HAMIS, confirming the glove's sensitivity in assessing fine finger motor dexterity (Table 4). Significant associations were also observed between HTS parameters and the mRSS.

**Table 4:** Correlation between HTS glove parameters with clinical and imaging findings in patients with SSc*

	Mean TD	Mean ITI	Mean MR
Age	r = 0.42, ***P* = 0.03**	r = 0.16, *P* = 0.43	r= 0.19, *P* = 0.34
Disease duration	r = 0.18, *P* = 0.37	r = 0.13, *P* = 0.50	r = 0.20, *P* = 0.35
DHI	r = 0.51, ***P* = 0.004**	r = 0.61, ***P* = 0.002**	r = 0.60, ***P* = 0.002**
HAMIS	r = 0.41, ***P* = 0.004**	r = 0.39, ***P* = 0.003**	r = 0.47, ***P* = 0.01**
SHAQ	r= 0.42, ***P* = 0.03**	r = 0.65, ***P* = 0.0004**	r = 0.56, ***P* = 0.003**
Mean grip strength test	r = −0.14, *P* = 0.5	r = −0.43, ***P* = 0.03**	r = 0.31, *P* = 0.13
Total mRSS	r = 0.27, ***P* = 0.04**	r = 0.4, ***P* = 0.03**	r = 0.42, ***P* = 0.03**
mRSS of right finger	r = 0.32, *P* = 0.12	r = 0.16, *P* = 0.06	r = 0.39, *P* = 0.07
mRSS of right hand	r = 0.3, ***P* = 0.04**	r = 0.33, ***P* = 0.03**	r = 0.38, *P* = 0.06
mRSS of left finger	r = 0.2, *P*= 0.37	r = 0.39, *P* = 0.09	r = 0.34, *P* = 0.10
mRSS of left hand	r = 0.2, *P*= 0.32	r = 0.35, ***P* = 0.02**	r = 0.31, *P* = 0.13
Dermal thickness of the left middle finger	r = −0.24, *P* = 0.32	r = −0.27, *P* = 0.25	r = 0.21, *P* = 0.37
Dermal thickness of the right middle finger	r = 0.21, *P* = 0.36	r = −0.22, *P* = 0.34	r = 0.14, *P* = 0.54
Dermal thickness of the dorsum of the left hand	r = 0.65, ***P* = 0.001**	r = −0.17, *P* = 0.45	r = −0.29, *P* = 0.20
Dermal thickness of the dorsum of the right hand	r = 0.66, ***P* = 0.001**	r = 0.16, *P* = 0.48	r = −0.3, *P* = 0.19
Total skin thickness	**r = 0.57, *P* = 0.007**	r = 0.32, *P* = 0.16	**r =** −**0.47, *P* = 0.031**
Mean capillary density at NVC	r = −0.03, *P* = 0.89	r = 0.01, *P* = 0.95	r = 0.02, *P* = 0.91
Rate of giant capillaries at NVC	r = −0.15, *P* = 0.46	r = 0.23, *P* = 0.28	r = 0.12, *P* = 0.58

*Note*: Significant‐values (*p* ≤ 0.05) are reported in bold.

* DHI, Duruöz Hand Index; HAMIS, Hand Mobility in Scleroderma; ITI, intertapping interval; MR, movement rate; mRSS, modified Rodnan skin score; NVC, nailfold videocapillaroscopy; SHAQ, Scleroderma Health Assessment Questionnaire; SSc, systemic sclerosis; TD, touch duration.

Interestingly, grip strength showed limited correlation with HTS metrics being correlated negatively only with the ITI. Notably, the mean TD positively correlated with dermal thickness of the dorsum of the hands measured by HFSU, as well as with total dermal thickness (all *P* < 0.05). Moreover, total skin thickness measured by HFSU showed a strong positive correlation with mean TD and a moderate negative correlation with MR, whereas no significant association was detected with mean ITI. No significant correlations were detected between HTS metrics and capillaroscopic parameters or disease duration.

Finally, SSc clinical subtype analyses based on clinical features, organ involvement, and autoantibody profile or NVC findings did not reveal significant differences in HTS parameters (Table 4 and Supplementary Table 2).

### Hand‐specific correlations of TD in patients with SSc

Because statistically significant differences were observed between the right and left hand for the TD parameter in patients with SSc (Supplementary Table 1), correlations were analyzed separately for each hand. TD of both the right and left hand showed significant correlations with disability questionnaires (DHI and SHAQ) and with the performance‐based hand mobility test (HAMIS). Additional significant associations emerged when TD was correlated with homolateral dermal thickness of the fingers and hand dorsum, as well as with total skin thickness measured by HFSU. Correlations between TD and total mRSS, capillaroscopic parameters (mean capillary density and rate of giant capillaries), and grip strength were nonsignificant (see Table 5 for details).

**Table 5:** Correlation between TD and traditional clinimetric parameters and imaging parameters in patients with SSc*

Traditional parameters	TD of right hand	TD of left hand
Age	**r = 0.43**, ***P* = 0.03**	**r = 0.41**, ***P* = 0.03**
Disease duration	r = 0.16, *P* = 0.25	r = 0.18, *P* = 0.13
DHI	**r = 0.53**, ***P* = 0.01**	**r = 0.55**, ***P* = 0.004**
HAMIS	**r = 0.55**, ***P* = 0.05**	**r = 0.53**, ***P* = 0.03**
SHAQ	**r = 0.37**, ***P* = 0.05**	**r = 0.38**, ***P* = 0.04**
Total mRSS	r = 0.3, *P* = 0.2	–
mRSS of right finger	r = 0.34, *P* = 0.09	–
mRSS of right hand	**r = 0.** **43, *P* = 0.03**	–
mRSS of left finger	–	r = 0.19, *P* = 0.37
mRSS of left hand	–	r = 0.17, *P* = 0.42
Grip strength right hand	r = 0.08, *P* = 0.70	–
Grip strength left hand	–	r = 0.17, *P* = 0.41
Dermal thickness of the left middle finger	–	**r = 0.46, *P* = 0.04**
Dermal thickness of the right middle finger	**r = 0.58, *P* = 0.006**	–
Dermal thickness of the dorsum of the left hand	–	**r = 0.57, *P* = 0.007**
Dermal thickness of the dorsum of the right hand	**r = 0.53, *P* = 0.013**	–
Total skin thickness	**r = 0.57, *P* = 0.007**	**r = 0.56, *P* = 0.008**
Mean capillary density at NVC	r = −0.08, *P* = 0.71	r = −0.02, *P* = 0.943
Rate of giant capillaries at NVC	r = −0.12, *P* = 0.58	r = −0.14, *P* = 0.5

*Note*: Significant‐values (*p* ≤ 0.05) are reported in bold.

* DHI, Duruöz Hand Index; HAMIS, Hand Mobility in Scleroderma; ITI, intertapping interval; MR, movement rate; mRSS, modified Rodnan skin score; NVC, nailfold videocapillaroscopy; SHAQ, Scleroderma Health Assessment Questionnaire; SSc, systemic sclerosis; TD, touch duration.

## DISCUSSION

This study demonstrated significant direct correlations between TD and dermal thickness measured by mRSS and HFSU, particularly at the dorsum of the hands and with the total skin thickness. These results suggest that functional hand impairment in patients with SSc seems to be closely linked to the degree of skin fibrotic involvement. In fact, HFSU, by quantifying dermal thickness, offers a valuable imaging biomarker that aligns well with the objective deficits measured by the engineered glove, underscoring the converging impact of skin fibrosis on dexterity outcomes.^28^, ^29^ Notably, the dermal thickness of the hand dorsum measured by HFSU showed stronger correlations with TD than local hand mRSS assessment, suggesting that ultrasonography may capture subtle skin changes more sensitively than clinical palpation.^27^, ^28^ Total mRSS significantly correlated with all three HTS parameters, indicating that the overall fibrotic burden also contributes to hand dexterity impairment.

The lack of correlation between glove parameters and other clinical factors, such as organ involvement, disease duration, and autoantibody subsets, indicates that hand dexterity impairment in SSc is relatively independent from these systemic features, at least in our sample. Regarding disease duration and autoantibody profile, no significant correlations were found between HTS parameters and disease duration, and subgroup analyses based on autoantibody subsets did not reveal significant differences. It is well recognized that specific autoantibody profiles, particularly anti‐RNA polymerase III, are associated with more rapid and extensive skin fibrosis and joint contractures, which could differentially impact hand dexterity, especially in later stages of dcSSc.^1^, ^30^ However, the small number of patients in each autoantibody subgroup in our pilot cohort limited the statistical power to detect such differences, and larger ongoing studies will specifically stratify patients by autoantibody profile and disease stage to clarify this issue.

This specificity underscores the glove's utility as a targeted tool for assessing hand function at any stage of the disease, rather than reflecting overall disease activity or severity. Patients with overlapping inflammatory arthritis were excluded from the present pilot study in order to assess the impact of skin fibrosis on hand dexterity. However, overlap syndromes and SSc‐associated arthritis are frequent and may represent a major contributor to hand disability in SSc.^30^, ^31^ Future studies including patients with and without articular involvement could leverage the HTS glove to differentiate the respective contributions of joint‐based and skin or tendon‐driven impairment to hand dysfunction, potentially guiding more targeted therapeutic strategies.

Of course, the hands in SSc may exhibit other local damages such as digital ulcers and joint contractures, resulting in decreased range of motion and significant pain.^30^, ^32^

Raynaud phenomenon itself, in acute phases, can exacerbate these issues by reducing blood flow to the fingers, leading to tissue sufferance and reduced function. Additionally, calcinosis, or the deposition of calcium in soft tissues, can cause further stiffness and discomfort. These manifestations collectively impair the functional abilities of the hands, making everyday tasks challenging and significantly impacting the quality of life of individuals with SSc.^33^ Therefore, all those conditions were excluded in the pilot study in order to focus on the pure skin fibrotic effect in limiting the hand dexterity. Although patients with digital ulcers were included in the cohort, all observed ulcers were small and located on the dorsal aspect of the fingers, a localization distinct from the more frequent ischemic fingertip ulcers and not directly interfering with the volar fingertip surface engaged during finger‐tapping. These factors, together with the small number of affected patients, may explain the lack of a significant impact of digital ulcers on HTS parameters and limited the statistical power to detect such an effect.

Although clinimetric tools such as the HAQ and DHI are widely used for RA, hand function assessment in SSc is frequently overlooked in comparison to RA, and similar standardized assessments for hand function in SSc, such as HAMIS test, are less commonly used but important for evaluating mobility limitations and disease progression in patients with SSc.^34^

In parallel, in the field of neurology, alongside traditional disability questionnaires, engineered glove‐derived parameters were used to assess hand and finger involvement in patients.^8^ Particularly, the HTS glove used in this study has been assessed in multiple sclerosis and Charcot‐Marie‐Tooth neuropathy to evaluate hand function.^11^

Indeed, recent studies conducted from our group demonstrated the HTS glove's effectiveness in assessing hand function in patients with RA.^9^, ^10^

The value of objectively and quantifiably assessing hand movement dexterity in patients with SSc is highlighted by the statistically significant differences observed in glove‐derived variables (MR, TD, and ITI) between patients with SSc and HCs, even among patients with minimal or no skin involvement as measured by the mRSS.

This concept appears to be supported by the significant correlation detected between HTS parameters and the SHAQ, DHI, and HAMIS test among patients with SSc. Of note, only TD, and not ITI or MR, showed significant interhand differences in patients with SSc, whereas all three parameters differed between hands in HCs. TD directly measures the contact time between fingers and therefore heavily depends on peripheral mechanical factors such as skin thickness, stiffness, and joint compliance. The selective interhand asymmetry for TD in patients with SSc may be consistent with the recent finding by Fourmond et al that the dominant hand in SSc exhibits significantly greater skin sclerosis and flexion deterioration than the contralateral hand, possibly due to increased mechanical stress exacerbating local fibrosis.^35^ The lack of significant interhand differences for ITI and MR in patients with SSc may also be partly attributable to the limited sample size, which could have reduced the statistical power to detect subtler asymmetries.

Specifically, reduced finger performance was closely associated with both greater perceived general disability and increased skin involvement. In fact, a negative correlation was observed between mRSS and the parameters of finger mobility.

In our study, the low‐grade correlation observed between HTS parameters and hand strength evaluation could be attributed to the fact that the HTS glove primarily assesses fine motor dexterity in hand movements, as opposed to strength, which is evaluated using the analogue dynamometer.

The absence of significant correlations between HTS parameters and NVC findings is an unexpected result of the present pilot study. Given that microvascular damage is a hallmark of SSc and progresses through well‐defined capillaroscopic patterns,^36^ a relationship between the severity of microangiopathy and hand dexterity impairment is biologically plausible. However, the uneven distribution of NVC patterns in our present cohort, with very few patients in the “early” pattern, severely limited the statistical power to detect such associations. Ongoing larger studies with a more balanced representation across NVC patterns will properly explore this relationship. Of particular interest, the HTS glove might be investigated in patients with very early SSc or pre‐SSc, where subtle hand dexterity changes detected by the glove might complement new and increasing capillaroscopic findings on this matter.^25^, ^37^, ^38^, ^39^ Overall, the HTS glove seems to offer an objective, noninvasive, safe, and rapid tool to evaluate hand function in patients with SSc, similar to its successful application in RA,^9^, ^10^ Given that preserving hand functionality should be one of the key goal in SSc treatment, the use of the HTS glove to assess hand disability, alongside traditional evaluation methods, offers valuable insights for a more comprehensive patient assessment.

Prospective investigations might correlate HTS glove parameters with SSc clinical changes over time in the same patients, as already achieved for hand assessments of patients with RA.^10^ Further studies we have planned will also aim to establish the minimal clinically important difference for HTS glove parameters in patients with SSc, in order to determine whether the differences detected by the glove are not only statistically significant but also clinically meaningful for patient management and therapeutic decision‐making. A potential learning effect with repeated use is minimized by the simplicity of the finger opposition task and the familiarization period before formal recording; moreover, our longitudinal RA study showed that HTS parameters worsened in patients with active disease despite repeated assessments, suggesting a negligible learning bias.^10^ Whether rehabilitation interventions such as occupational or physical therapy can improve HTS parameters in patients with SSc remains a clinically relevant question for future interventional studies. From a clinical perspective, the HTS glove is not intended to replace tools such as HFSU or PROMs, but rather to complement them. Unlike SHAQ, DHI, and HAMIS, which rely on subjective perception or semiquantitative observer scoring, the glove provides objective, continuous numerical data on hand dexterity, more sensitive to subtle longitudinal changes. HFSU measures a different construct (dermal thickness), whereas the glove captures its functional consequence on finger movements; the significant correlations between TD and HFSU‐measured dermal thickness confirm that these tools offer converging but complementary information. Practically, the entire HTS assessment, including a familiarization period of approximately 2 to 3 minutes and the formal 20‐second recording for each hand, can be completed in approximately 5 minutes per patient. The test is noninvasive, requires minimal operator training, and has a low cost compared with ultrasonographic equipment, making it feasible for routine clinical use and multicenter studies.

However, the current study displays multiple limitations: first, the small number of patients with SSc and HCs enrolled may have concealed some interesting correlations worth analyzing. Although the age difference between patients with SSc and HCs was not statistically significant, the correlation between age and TD observed in patients with SSc suggests that age may act as a contributing factor, and future studies should consider age‐matched stratification or multivariate adjustment.

Indeed, a larger patient sample could have allowed a more robust division of patients with SSc into clinical SSc subtypes based, for example, on limited or diffuse skin involvement, but also concerning autoantibody complex profiles and the NVC pattern scores.

Despite the relevance of microvascular evaluations such as NVC quantification in SSc, the limited sample size may have reduced the statistical power to detect significant correlations with HTS parameters.

Furthermore, another limitation is the time gap of up to three months between the administration of the HTS glove test and the collection of other clinical data, including the mRSS. Because SSc can present dynamic clinical fluctuations, this interval might introduce variability in the clinical parameters compared to the exact moment of the hand dexterity assessment. However, the actual mean interval was considerably shorter than the maximum allowed window (42 days for the mRSS and approximately 60 days for the other clinical and imaging parameters), which likely mitigated this variability. Finally, as all clinimetric and functional assessments were conducted by a single, unblinded operator, a potential detection bias cannot be entirely excluded.

Nevertheless, the study exhibits several strong points. Most notably, this is the first investigation applying an engineered sensor glove for the quantitative assessment of hand dexterity in patients with SSc, introducing a rapid, noninvasive, and reliable modality for objective outcome measurement.

Of note, the significant correlation observed between the HTS parameters with the mRSS and the ultrasonography‐verified hand dermal thickness represents an innovative convergence of functional and imaging assessment, enhancing the repertoire of safe and noninvasive tools available for clinical and research evaluation.^40^

Therefore, further longitudinal studies are ongoing to explore the potential of the HTS glove in monitoring and quantifying hand and systemic skin progression in SSc, as well as in assessing response to established and emerging therapies, ultimately supporting personalized, safe, and easily applicable strategies to limit disability and improve patients’ quality of life.

## AUTHOR CONTRIBUTIONS

All authors contributed to at least one of the following manuscript preparation roles: conceptualization AND/OR methodology, software, investigation, formal analysis, data curation, visualization, and validation AND drafting or reviewing/editing the final draft. As corresponding author, Prof. Cutolo confirms that all authors have provided the final approval of the version to be published and takes responsibility for the affirmations regarding article submission (eg, not under consideration by another journal), the integrity of the data presented, and the statements regarding compliance with institutional review board/Declaration of Helsinki requirements.

## Supporting information


**Disclosure Form**:

**Supplementary Table 1.** Inter‐hand comparison in systemic sclerosis patients and healthy controls. Abbreviations. HCs: healthy controls, SSc: systemic sclerosis.
**Supplementary Table 2**. Analyses of glove parameters according to the clinical features and autoantibody profile **of patients with SSc** clinical subtypes.
