# Supplementary material for: An Engineered Glove for the Objective Assessment of Hand Dexterity in Patients With Systemic Sclerosis
Source: ACR Open Rheumatol. 2026 Jul 3;8(7):e90082. doi: 10.1002/acr2.90082 (PMC13329684; doi:10.1002/acr2.90082)
Supplement: Supplementary file 2 — Supplementary Table 1. Inter‐hand comparison in systemic sclerosis patients and healthy controls. Abbreviations. HCs: healthy controls, SSc: systemic sclerosis. Supplementary Table 2. Analyses of glove parameters according to the clinical features and autoantibody profile of patients with SSc clinical subtypes. [file ACR2-8-e90082-s001.docx]

**Supplementary materials**

|  | **TD of the left hand**  **(mean ± SD)** | **TD of the right hand**  **(mean ± SD)** | **p-value** | **ITI of the left hand**  **(mean ± SD)** | **ITI of the right hand**  **(mean ± SD)** | **p-value** | **MR of the left hand**  **(mean ± SD)** | **MR of the right hand**  **(mean ± SD)** | **p-value** |
| --- | --- | --- | --- | --- | --- | --- | --- | --- | --- |
| **SSc patients** | 298,98 ± 139,97 | 283,35 ± 139,49 | **0.02** | 511,93 ± 325,97 | 504,63 ± 379,83 | 0.66 | 1,57 ± 0,69 | 1,61 ± 0,68 | 0.78 |
| **HCs** | 121,28 ± 33,08 | 101,32 ± 20,27 | **0.01** | 102,97 ± 26,81 | 123,67 ± 35,18 | **0.02** | 3,69 ± 0,64 | 4,11 ± 0,59 | **0.02** |

**Supplementary Table 1.** Inter-hand comparison in systemic sclerosis patients and healthy controls. Abbreviations. HCs: healthy controls, SSc: systemic sclerosis.

**Abbreviations.** HCs: healthy controls, SD: standard deviation

| **Clinical features** | | | |
| --- | --- | --- | --- |
| **HTS glove parameters** | **lcSSc** | **dcSSc** | **p-value** |
| TD (ms) | 274.13 ± 110.07 | 332.8 ± 195.92 | 0.35 |
| ITI (ms) | 502.41 ± 331.86 | 536.41 ± 334.68 | 0.82 |
| MR (Hz) | 1.62 ± 0.66 | 1.51 ± 0.75 | 0.71 |
|  | **Patients with ILD** | **Patients without ILD** |  |
| TD (ms) | 296.89 ± 151.8 | 280.28 ± 117.07 | 0.80 |
| ITI (ms) | 504.7 ± 249.73 | 526.05 ± 508.17 | 0.89 |
| MR (Hz) | 1.58 ± 0.67 | 1.66 ± 0.78 | 0.80 |
|  | **Patients with esophageal involvement** | **Patients without esophageal involvement** |  |
| TD (ms) | 277.55 ± 118.15 | 309.17 ± 166.98 | 0.59 |
| ITI (ms) | 536.76 ± 385.66 | 480.39 ± 273.1 | 0.69 |
| MR (Hz) | 1.62 ± 0.75 | 1.57 ± 0.64 | 0.85 |
|  | **Patients with PAH** | **Patients without PAH** |  |
| TD (ms) | 272.09 ± 123.86 | 294.89 ± 144.95 | 0.80 |
| ITI (ms) | 699.77 ± 425.97 | 483.95 ± 321.28 | 0.30 |
| MR (Hz) | 1.31 ± 0.55 | 1.64 ± 0.71 | 0.45 |
|  | **Patients with active digital ulcers** | **Patients with active digital ulcers** |  |
| TD (ms) | 271.8 ± 108.21 | 298.79 ± 151.55 | 0.69 |
| ITI (ms) | 455 ± 156.83 | 529.57 ± 375.86 | 0.64 |
| MR (Hz) | 1.63 ± 0.39 | 1.59 ± 0.77 | 0.92 |
|  | **Patients with kidney involvement** | **Patients without kidney involvement** |  |
| TD (ms) | 298.93 ± 141.31 | 290.67 ± 143.67 | 0.92 |
| ITI (ms) | 384.92 ± 141.38 | 536.13 ± 356.4 | 0.42 |
| MR (Hz) | 1.68 ± 0.76 | 1.58 ± 0.69 | 0.81 |
| **Autoantibody profile** | | | |
|  | **ACA positive** | **ACA negative** | **p-value** |
| TD (ms) | 264.07 ± 104.97 | 314.5 ± 154.91 | 0.39 |
| ITI (ms) | 521.43 ± 449.1 | 527.99 ± 242.81 | 0.96 |
| MR (Hz) | 1.67 ± 0.74 | 1.47 ± 0.58 | 0.47 |
|  | **Scl70 positive** | **Scl70 negative** |  |
| TD (ms) | 255.32 ± 100.6 | 324.36 ± 156.77 | 0.23 |
| ITI (ms) | 462.03 ± 125.76 | 570.88 ± 413.96 | 0.37 |
| MR (Hz) | 1.61 ± 0.44 | 1.49 ± 0.76 | 0.67 |

**Supplementary Table 2.** Analyses of glove parameters according to the clinical features and autoantibody profile **of patients with SSc** clinical subtypes.

**Abbreviations in alphabetical order:** ACA = anti-centromere antibodies; dcSSc = diffuse cutaneous systemic sclerosis; HTS glove = Hand Test System glove; ILD = interstitial lung disease; lcSSc = limited cutaneous systemic sclerosis; MR = movement rate (Hz); PAH = pulmonary arterial hypertension; Scl70 = anti-topoisomerase I antibodies; TD = touch duration (ms); ITI = inter-tapping interval (ms).
